# Efficient Separation of a Novel Microbial Chassis, *Vibrio natriegens*, from High-Salt Culture Broth Using Ceramic Ultrafiltration Membranes

**DOI:** 10.3390/membranes15040121

**Published:** 2025-04-11

**Authors:** Stefan Schwarz, Rong Fan, Mehrdad Ebrahimi, Peter Czermak

**Affiliations:** 1 Institute of Bioprocess Engineering and Pharmaceutical Technology, University of Applied Sciences Central Hesse, 35390 Giessen, Germany; stefan.schwarz@lse.thm.de (S.S.); fanrong@mail.ipe.ac.cn (R.F.); mehrdad.ebrahimi@lse.thm.de (M.E.); 2 State Key Laboratory of Biopharmaceutical Preparation and Delivery, Institute of Process Engineering, Chinese Academy of Sciences, Beijing 100190, China; 3 School of Chemical Engineering, University of Chinese Academy of Sciences, Beijing 100190, China; 4 Faculty of Biology and Chemistry, Justus-Liebig-University Giessen, 35390 Giessen, Germany

**Keywords:** fouling, *Vibrio natriegens*, compressibility index, resistance in series model, Hermia’s laws, ceramic membranes, ultrafiltration, CFF

## Abstract

*Vibrio natriegens* is widely used as a production host for biotechnological processes due to its superior maximum glucose consumption rate, high growth rate, and abundant ribosomes. Most bioprocesses also need a scalable biomass separation step. This can be achieved by cross-flow filtration with ceramic membranes, although the membrane pores are susceptible to fouling. However, the fouling characteristics of *V. natriegens* culture broth have not been investigated in detail. We therefore characterized membrane fouling during the separation of *V. natriegens* biomass from culture broth using a cross-flow filtration plant with ceramic membranes. The resistance in series model was used to quantify the fouling-induced resistance caused by the different components of the culture broth. The total fouling resistance was 4.1·10^9^ ± 0.6·10^9^ m^−1^ for the culture broth and 5.4·10^9^ ± 0.7·10^9^ m^−1^ for the summed broth components. Reversible resistance accounted for 86% and 81% of these totals, respectively. We then applied Hermia’s adapted filtration laws to determine the dominant fouling mechanism induced by the different broth components. In a further step, we established a setup to determine the compressibility index of the cells during cross-flow filtration, resulting in an estimated value of 0.55 ± 0.04. These results will facilitate the design of economic filtration plants and will help to establish *V. natriegens* as a production host for large-scale industrial processes.

## 1. Introduction

*Vibrio natriegens* is a rod-shaped, halophilic, Gram-negative marine bacterium with an approximate size of 3.5 × 0.3 µm [1,2]. Its superior maximum glucose consumption rate and high growth rate make it useful for many biotechnological applications, which are facilitated by its compatibility with the plasmid systems originally developed for *Escherichia coli* [3,4,5,6]. Accordingly, *V. natriegens* has been developed as an expression host for the production of recombinant peptides and proteins and has also been used to develop a cell-free expression system and functional nanoparticles [7,8,9,10,11,12,13,14,15]. Owing to its high salt tolerance, *V. natriegens* can be cultivated under a non-sterile condition, thus decreasing the operating costs for sterilization in large-scale industrial processes. As a promising chassis in synthetic biology, *V. natriegens* is able to secrete organic alcohols, acids, and heterologous proteins as products into the extracellular space. Like other cell-based expression systems, it is necessary to separate the *V. natriegens* biomass from the culture broth at the beginning of downstream processing. This can be achieved by cross-flow filtration with ceramic membranes, but the fouling characteristics of the culture broth have not been investigated in detail, and this is the starting point for process optimization.

Ceramic membranes are widely used for the filtration of organic process streams in the food and feed, pulp and paper, and bulk chemical industries, as well as in the biotechnology sector for the purification of proteins [16,17,18,19,20,21,22]. The advantages of ceramic membranes include their resistance to harsh conditions, such as extreme pH and temperatures, as well as chemical cleaning agents. This means they can be regenerated frequently, which is beneficial because membrane fouling is a major limitation to the efficiency of filtration processes. Other advantages include their high resistance to abrasive media, their longevity, and their high flux per unit area, which combine to make them more attractive from an economic perspective [23,24,25]. Owing to their high chemical stability, ceramic membranes are suitable to separate *V. natriegens* with the products from a high-salt-containing medium. However, this hypertonic environment may change the surface interaction between biological particles and membrane materials and cause severe fouling, which changes the separation efficiency and selectivity. Therefore, we systematically analyzed the fouling mechanism during the filtration of *V. natriegens* in a hypertonic solution with ceramic membranes.

The flux through a membrane depends on the driving force, the total resistance, and the permeate viscosity. The driving force during filtrations with porous membranes is the transmembrane pressure. The total resistance is composed of the membrane resistance and the fouling resistance. The membrane resistance can be calculated using Equation (1), which is based on Darcy’s law [26,27]. The membrane resistance is equal to the total resistance when the permeate is pure water passing through a clean membrane.

(1) $$R_{m}=R_{T}=\frac{∆p}{µ*J}$$

where *R_m_* is the membrane resistance, *R_T_* is the total resistance, ∆*p* is the transmembrane pressure, *µ* is the dynamic viscosity, and *J* is the filtrate flux.

During filtration, a filter cake builds up on the retentate side of the membrane, and the total resistance is therefore the sum of the membrane resistance and cake resistance. In this case, the flux can be calculated using Equation (2) [28].

(2) $$J=\frac{\Delta p}{µ\cdot (\alpha \cdot w+R_{m})}$$

where *α* is the specific cake resistance and *w* is the weight of the cake per filtration area. The specific cake resistance is calculated using Equation (3), which is a rearrangement of Equation (2).

(3) α=ΔpJ·µ−Rmw

We can differentiate between the true specific cake resistance and the apparent specific cake resistance; the latter is shown in Equation (3). The true specific cake resistance is calculated by replacing *R_m_* in Equation (3) with *R_mi_*, the sum of pristine membrane resistance and resistance caused by the fouling inside pores, resulting in Equation (4) [26]. Several approaches have been described to determine the cake mass. In addition to weighing the entire membrane or the mechanically removed cake, it is also possible to remove the membrane and digest the cake to deduce the cake mass from the amount of protein released [29,30,31]. Another approach, which makes it possible to determine the cake mass without removing the membrane, is to measure the biomass in the feed and calculate the cake mass as the difference before and after filtration [26].

(4) α=ΔpJ·µ−Rmiw

**Figure 1:**
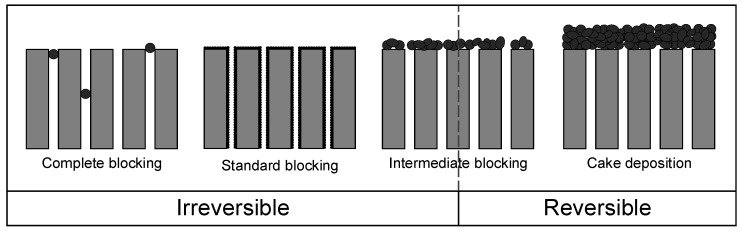
Visualization of different fouling mechanisms, adapted from Di Bella and Di Trapani (2019) [27].

To understand *R_mi_*, it is necessary to know that the resistances that lead to membrane fouling can be divided into those reflecting reversible and irreversible fouling. Reversible fouling is the portion of fouling that can be removed by mechanical cleaning, which can involve procedures such as backwashing, gas scouring, and relaxation, whereas irreversible fouling cannot be removed. The irreversible fouling resistance of a membrane is determined by passing water through a mechanically cleaned membrane. The value is then calculated using Equation (1) but replacing *R_m_* with *R_mi_*. The reversible fouling resistance *R_rf_* is the difference between the total resistance of the fouled membrane minus the resistance of the cleaned membrane and the irreversible fouling resistance *R_if_*, resulting in *R_mi_* and leading to Equation (5) [25,27,32,33]. The distinction between reversible and irreversible fouling allows us to assign specific fouling mechanisms to each of the resistances. Irreversible resistance is caused by the fouling mechanisms of complete blocking, standard blocking, and, in part, intermediate blocking, whereas reversible resistance is caused by cake deposition combined with intermediate blocking (Figure 1).

In addition to the aforementioned definitions, another definition of reversible fouling includes all fouling that can be removed mechanically and chemically, whereas irreversible fouling includes fouling that cannot be removed by these means [34,35]. These alternative definitions are not used in the current article.

(5) $$R_{T}=R_{m}+R_{if}+R_{rf}$$

With increasing transmembrane pressure, the specific cake resistance also increases. This can be mathematically expressed using Equation (6), where *α*′ is a coefficient and *n* is the compressibility index [28,36,37]. Several factors affect the compressibility index, including cell shape and size, as well as the ionic strength of the feed [28,38,39]. Ellipsoidal cells tend to have lower compressibility indices than rod-shaped cells [28,39].

(6) $$\alpha =\alpha^{\prime }\cdot {\left(\Delta p\right)}^{n}$$

The rearrangement of Equation (6) gives Equation (7). These equations can be solved for *n* by measuring *α* at two different transmembrane pressures (Equation (8)).

(7) $$\alpha^{\prime }=\frac{\alpha }{{\left(\Delta p\right)}^{n}}$$

(8) $$n=\log_{\frac{{∆p}_{2}}{{∆p}_{1}}}\left(\frac{\alpha_{2}}{\alpha_{1}}\right)$$

The term ∆*p* is often used for the transmembrane pressure [28,38,40], but the force driving compression is not transmembrane pressure but the proportion of the pressure that falls on the cake [41]. This proportion depends on the individual resistances of the cake and the membrane, and it changes with the membrane resistance if the cake resistance remains constant. For this reason, we prefer to use the pressure drop across the cake to calculate the compressibility index so that it can be considered and compared independently of the membrane [42]. The pressure drop over the cake is calculated using Equation (9).

(9) $${∆p}_{c}=∆p-µ\cdot J\cdot R_{mi}$$

There are two popular approaches to describe fouling mechanisms. One is the resistance in series model, which distinguishes components in the feed and allocates to each a share of the total resistance. Equation (10) formalizes this approach [43,44]. According to this model, the sum of the fouling resistances of the medium (*R_ME_*), cells (*R_C_*), and byproducts of microbial activity (*R_P_*) is equal to the fouling resistance of the fermentation broth.

(10) $$R_{FB}=(R_{ME}+R_{C}+R_{P})$$

The other approach distinguishes between the fouling mechanisms of complete blocking, standard blocking, intermediate blocking, and cake deposition. The mathematical description of this model for dead-end filtration is based on Equation (11) [27,45,46]. The mechanisms underlying the different types of fouling (and their reversibility, if appropriate) are summarized in Figure 1.

(11) $$\frac{d^{2}t}{dV^{2}}=k{\left(\frac{dt}{dV}\right)}^{n}$$

The different fouling mechanisms are also known as Hermia’s laws [47], and the corresponding Equations (12)–(15) are presented in Table 1.

This model was adapted to cross-flow filtration by including the influence of convective transport on the fouling layer (Equation (16)). Equations (17)–(20) in Table 2 distinguish the different fouling mechanisms [47,48]. In comparison to the other mechanisms, standard blocking is assumed to take place generally inside the membrane pores; this fact is dominated by the diffusion of solutes passing through the membrane pores. It suggests that standard blocking is not influenced by the cross-flow rate.

(16) $$-\frac{dJ}{dt}(J^{n-2})=k(J-J_{ss})$$

## 2. Materials and Methods

### 2.1. Production of Fermentation Broth

We cultivated *V. natriegens* Vmax cells (Synthetic Genomics, Calipatria, CA, USA) in 250 mL of the recommended medium [7] in 1 L baffled shake flasks (Schott, Mainz, Germany). The medium was inoculated at an OD_600_ of 0.03 and cultivated overnight in a Multitron shaking incubator (Infors, Bottmingen, Switzerland) set at 32 °C and 250 rpm.

### 2.2. Feed Solutions for Filtration with the Ceramic Membrane

The feed solutions tested in the resistance in series model were *V. natriegens* cells in 3% NaCl, medium, culture supernatant, and culture broth. The medium deviated from the medium used for fermentation by excluding glucose. All feed solutions were adjusted to pH 7.4 ± 0.1 using 1 M NaOH or 1 M HCl. To adjust the OD_600_ of the culture broth, a portion was centrifuged at 8000 g for 10 min, and the culture broth was diluted with the supernatant to an OD_600_ of 7.4. To prepare the cell solutions in 3% NaCl, 1.4 L of the overnight culture was centrifuged as described above, and the pellets were resuspended in 600 mL of 3% NaCl and then centrifuged again. The pellets were then resuspended in 3% NaCl, and the suspension was diluted in 3% NaCl until the OD_600_ was 7.4 ± 5% (for the fouling models) or 0.95 ± 5% (for the compressibility index experiments). The supernatant was generated by single-step centrifugation of the overnight culture, as described above, followed by passage through a 0.2 µm bottle-top filter (VWR, Radnor, PA, USA).

### 2.3. Filtration Plant

**Figure 2:**
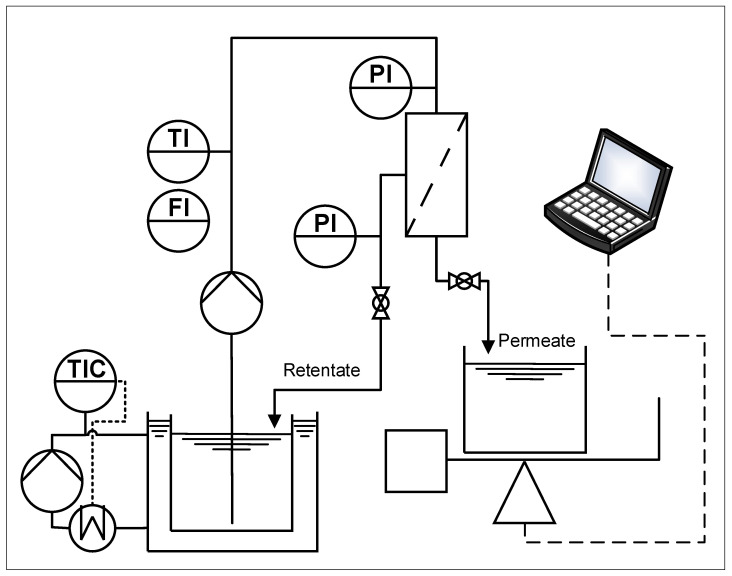
Setup of the filtration plant: PI = pressure indicator, TI = temperature indicator, FI = flow indicator, and TIC = temperature indicator and controller.

The filtrations tested using the resistance in series model and the fits using Hermia’s laws were based on a UF50A membrane (Atech Innovations, Gladbeck, Germany) with a 50 nm cut-off, an inner diameter of 6 mm, and an effective length of 23.5 cm, resulting in a filtration area of 0.0044 m^2^. The filtrations for the compressibility index tests were carried out using the same type of membrane, an effective length of 45 cm, an outer diameter of 25.4 cm, and 19 channels, each with a diameter of 3.3 mm, resulting in a filtration area of 0.089 m^2^. The membrane materials and support materials consisted of Al_2_O_3_. The construction of the filtration plant is shown in Figure 2. The membrane was inserted into a stainless-steel holder, and the feed from the tempered double-jacket reservoir was directed through an SM6000 flow sensor (IFM, Essen, Germany) to the membrane via an FCPA 80B-4/HE rotary vane pump (AFT, Rosstal, Germany). The feed temperature was adjusted by cooling the reservoir using an ECO RE 420 thermostat (Lauda, Lauda-Koenigshofen, Germany). Pressure sensors were located before and behind the membrane. The pressure sensor behind the membrane was adjacent to a ball valve, allowing the transmembrane pressure to be adjusted. Behind the ball valve, the feed was recirculated back to the feed reservoir. On the permeate side of the membrane, the permeate was fed into a beaker via a pressure sensor and a downstream ball valve. The beaker was placed on a PFB 3000-2 electrical scale (Kern & Sohn, Balingen-Frommern, Germany) linked to a laptop. The permeate flow was thus recorded by averaging over 10 s using LabVision (HiTec Zang, Herzogenrath, Germany). The permeate in the beaker was emptied back into the permeate reservoir before the mass in the beaker exceeded 10% of the feed mass.

### 2.4. General Procedure for the Filtration Experiments

Before each filtration experiment, the cleaning solution in the filtration system was replaced with water until the permeate pH became neutral. The membrane resistance was then determined by measuring the permeate flux at five equidistant transmembrane pressures from 0.4 to 2.0 bar, using Equation (1). In the next step, the water was drained and replaced with 3% NaCl. Afterward, a feed volume of 1200 ± 100 mL was filled into the system and circulated for 10 min under filtration conditions with a closed permeate valve. Filtrations for the resistance in series model were carried out for a minimum of 90 min or until a steady-state transmembrane flux was established, whereas filtrations to determine the compressibility index were completed after 20 min. For each filtration testing the resistance in series model, a transmembrane pressure of 0.8 bar was applied, while the temperature was maintained at 25 ± 1 °C. For the single-channel membrane, we used a cross-flow velocity of 3 m·s^−1^, whereas a higher volumetric flow, equivalent to a flow velocity value of 0.5 m·s^−1^, was used for the 19-channel membrane. This corresponds to a wall shear rate of approximately 1212 s^−1^ for the 19-channel membrane (3.3 mm channel diameter) and 4000 s^−1^ for the single-channel membrane (6 mm channel diameter), calculated based on the respective flow velocities. Samples were taken from the permeate and feed to determine the protein concentration using a Bradford assay, as described by Schwarz et al. [7]. After filtration, we evaluated the viscosity of a permeate sample using an MCR102 modular compact rheometer (Anton Paar, Ostfildern-Scharnhausen, Germany). The feed was drained and replaced with at least 1 L of water, which was circulated within the filtration plant at a cross-flow velocity of 4.5 m·s^−1^ (single-channel membrane) or 0.8 m·s^−1^ (19-channel membrane) for 15 min with a closed permeate valve. The water was then replaced, and the procedure was repeated with an open permeate valve. Having replaced the feed with fresh water again, the resistance of the irreversibly fouled membrane was determined as described above.

### 2.5. Filtration Experiments to Determine the Compressibility Index

The general procedure described above was followed with the following deviations. After rinsing the cleaning solution, the water was replaced with 3% NaCl. The cross-flow velocity was set to 0.5 m·s^−1^, and the transmembrane pressure was set to the value used for subsequent filtration. The feed was circulated in the filtration system with the permeate valve closed using the specified parameters for 10 min before filtration was initiated by opening the permeate valve. To avoid any influence on the biomass concentration in the feed, the permeate flow was only determined at the end of the filtration; otherwise, the permeate was returned directly to the feed reservoir. Five filtrations were performed at transmembrane pressures of 0.4–2.0 bar in equidistant steps. A feed sample was taken every minute to determine the OD_600_. At the end of filtration, the permeate flux was measured three times, and the viscosity of a permeate sample was determined as described above.

### 2.6. Chemical Cleaning of Ceramic Membranes

The membranes were cleaned at the same cross-flow velocities applied during filtration and at a transmembrane pressure of 0.4 bar. The first cleaning solution was 8 g·L^−1^ Dismozon (Hartmann, Heidenheim, Germany), applied for 1 h. The second cleaning solution was 1% (*w*/*v*) citric acid (Carl Roth, Karlsruhe, Germany), applied for 1 h. The last cleaning solution was 1% (*w*/*v*) P3 Ultrasil 14 (Ecolab Deutschland, Monheim am Rhein, Germany), applied for 2 h and also used for the subsequent storage of the filtration plant.

### 2.7. Data Analysis

The resistances of the clean and irreversibly fouled membranes were calculated using Equation (1). The water fluxes of the clean membrane were used to calculate the membrane resistance. The total fouling resistance was calculated using the flux of the fouled membrane minus the value generated with the clean membrane. The reversible fouling resistance was the difference between the total fouling resistance and the irreversible resistance. The irreversible fouling resistance was the resistance of the mechanically cleaned membrane minus the resistance of the clean membrane. To compare resistances for the resistance in series model, total fouling resistance values were calculated from the flux 90–95 min after the start of filtration. All fitting was carried out using Origin (Origin Lab, Northampton, MA, USA). The initial value for the compressibility index fit was calculated using Equation (8). The corresponding specific cake resistances were calculated using Equations (6) and (9). The cake mass was calculated using the OD_600_ values. Fouling analysis according to Hermia’s laws was carried out using Equations (12)–(20).

## 3. Results

### 3.1. Resistance in Series Model

**Figure 3:**
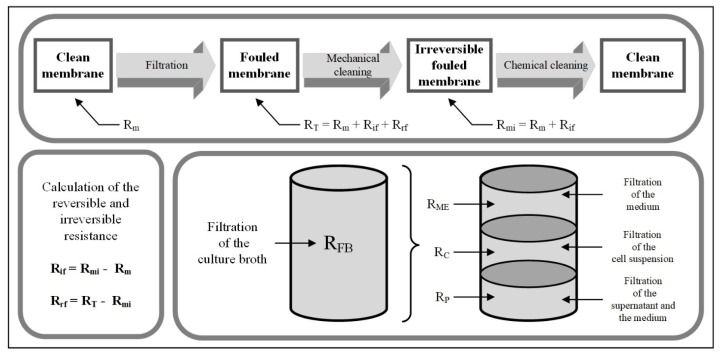
Overview of the procedure for calculating the resistances of the resistance in series model.

The resistance in series model was used to determine the contribution of different components in the culture broth to the fouling resistance. The resistances of the medium, the cell suspension, and the culture broth were determined directly via the permeate flows before and after filtration, as well as after mechanical cleaning, as described in the Materials and Methods Section. The resistances of the microbial byproducts result indirectly from the subtraction of the resistances of the supernatant and the medium. Figure 3 provides an overview of the procedure for collecting the necessary data.

Microbial culture broth is a complex mixture consisting of cells, cell debris, released biomacromolecules, and small molecular substances. Owing to the difference in size and surface chemistry, these bioparticles may exhibit various transmembrane manners. Our previous study demonstrated that large particles like cells can be completely rejected by a 50 nm ceramic membrane, while proteins were partially adsorbed in the inner pores of the membranes. The content of these components alters the flux and predominant fouling mechanism during the filtration. In order to figure out the individual influence of each factor on fouling formation, we analyzed the fouling resistances by filtrating different bio-fluids step by step.

**Figure 4:**
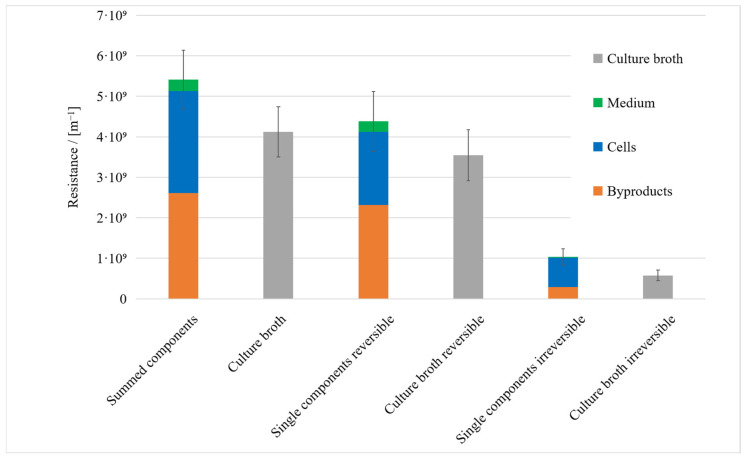
Resistances of broth components compared to the overall resistance of the culture broth. Error bars were calculated by Gaussian error propagation.

**Figure 5:**
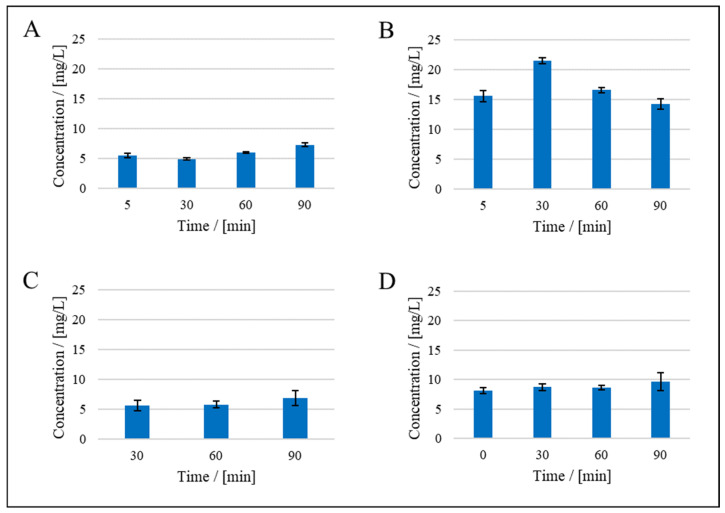
Protein concentration in the feed and permeate: (**A**) = cells, permeate side, (**B**) = culture broth, permeate side, (**C**) = supernatant, permeate side, and (**D**) = supernatant, feed side. Data are means ± standard deviations (n = 3).

Microbial byproducts accounted for the largest share of the total resistance (48%), followed by the cells (47%) and the medium (5%). The sum of these individual resistances was compared to the resistance of the culture broth, and the same comparison was carried out for the reversible and irreversible resistances. For the total resistance and reversible resistance, the sum of the individual resistances overlapped with that of the culture broth when taking experimental uncertainty into account, but for the irreversible resistance, the sum of individual resistances was higher than that of the culture broth (Figure 4). This can be explained by cell lysis in NaCl during filtration. We compared the OD_600_ of cell suspensions in 0.9% and 3% NaCl solutions. It remained stable in the higher concentration of NaCl but gradually declined in the 0.9% NaCl solution, suggesting cell lysis and loss in viability caused by low osmolarity. The osmolarity in the medium is 1.41 mol/L, and the osmolarity of 30 g/L NaCl is lower at 1.03 mol/L. A solution with 9 g/L NaCl has an even lower osmolarity of 0.34 mol/L. All microorganisms, except perhaps halophilic *Halobacteria*, have a positive turgor. In order to maintain this osmotic pressure, they must adapt the osmolarity of the cell interior to their environment. To this end, bacteria vary the concentration of salts and compatible solutes inside the cell [49]. *Vibrionaceae*, which include *Vibrio natriegens*, are able to adapt their osmolarity through the uptake and synthesis of compatible solutes [50]. If the osmolarity in the environment decreases faster than in the cell, the turgor increases, which can lead to a weakening of the cell and disruption [51]. Another possible explanation is the absence of substrates or the combination of both circumstances, considering that the transport of compatible solutes across the cell wall is partly dependent on ATP. The protein concentration in the cell suspension increased between the 30 min and 90 min time points during filtration, while the reverse was observed during the filtration of culture broth (Figure 5). Here, a notable fact is that the protein concentration in the permeate of the culture broth was higher than it was in the feed of cell-free supernatant, suggesting a release of protein from cells during the filtration of the culture broth (Figure 5B,D). We assume that the harsh conditions within the filtration system led to the desorption of the cell-bound proteins that remained associated with the cells during the preparation of the supernatant. This hypothesis is further supported by the significant protein concentration observed in the permeate during the filtration of cells suspended in a NaCl solution.

Considering the biological effects described above, the lysis of *V. natriegens* observed in the preliminary experiments (see the supplemental data) led to our hypothesis that cell debris was the cause of the increase in irreversible fouling. The decreasing protein concentration in the culture supernatant may reflect the adsorption of proteins onto the membrane and/or depletion of the proteins due to the metabolic activity of *V. natriegens*. Members of the genus *Vibrio* are known to secrete metalloproteases and serine proteases [52]. In addition, numerous amino acids are among the substrates that can be utilized by *V. natriegens* [53]. This may also explain why this continuous degradation did not occur in the cell suspension. Due to the cell separation and subsequent wash step, the proteases were separated from the cell suspension. The constant protein concentration during the filtration of the supernatant excludes the first hypothesis, making the second the more likely explanation. The protein concentration in the permeate of the supernatant filtration is lower than in the feed. We assume that this is due to the fact that some of the proteins are retained by the membrane. This conclusion is also consistent with the observation that in the case of the microbial byproducts, a part of the fouling resistance is of a reversible nature. The initial increase in the protein concentration in the culture broth in the period of 5 to 30 min after the start of filtration may be a result of cell lysis caused by the shear forces in the pump. This increase cannot be observed in the cell suspension. This can be attributed to the shear forces during the centrifugation of the cells when preparing the cell suspension. As a result, a more shear-sensitive proportion of the bacterial population may have already been lysed before the cell suspension was used in the filtration system.

### 3.2. Compressibility Index

The compressibility index was calculated using shorter filtration periods to ensure that cell lysis had a negligible impact on the fouling behavior. In the preliminary experiments, the OD_600_ of the feed declined while circulating in the filtration system. We assume this was caused by cell lysis during filtration under suboptimal conditions, which increased the degree of irreversible fouling by the cell lysate. This is supported by the increasing protein concentration between the 30 min and 90 min time points (Figure 5). The preliminary experiments revealed that compressibility was affected by the culture age and NaCl concentration. Therefore, cells were taken from fresh overnight cultures, and we set the NaCl concentration to 30 g·L^−1^ in the feed. The filtration time was also reduced to 20 min, resulting in a smaller proportion of irreversible fouling relative to the total fouling resistance (1–3%). In the longer filtration runs for the other fouling models, the proportion of the irreversible fouling resistance was 29%. The proportion of irreversible fouling in total fouling resistance appears to be variable. A closer examination with the aid of the errors reveals that the irreversible resistance at 1.6 bar cannot be explained by the errors (see the supplemental data). We assume that this is an outlier.

**Figure 6:**
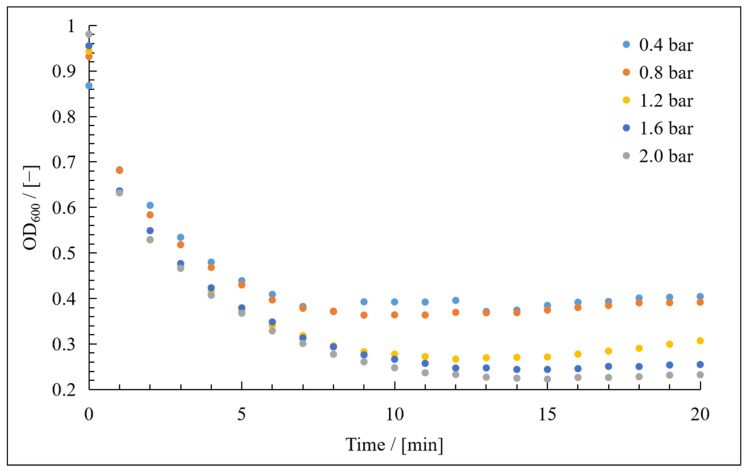
OD_600_ values measured during the course of filtration to determine the compressibility index.

**Figure 7:**
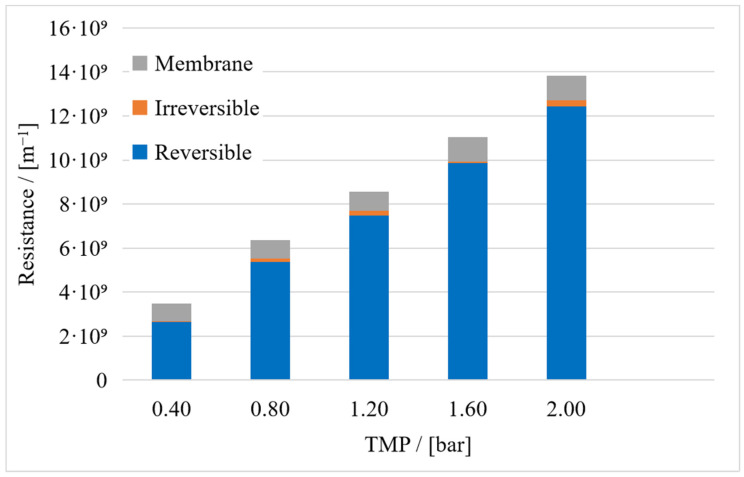
Components of total resistance to calculate the compressibility index (resistance of the cleaned membrane, and resistances attributed to reversible and irreversible fouling).

The measurement of cell mass in the feed during these experiments ensured that an equilibrium was reached between the mass of the cells in the feed and in the cake on the membrane ~15 min after the start of filtration (Figure 6). Similar findings, in which an equilibrium was established within 10–15 min, were reported in another study [26]. The authors also observed continual flux decline after cake formation, which agrees with our observation of irreversible fouling (Figure 7). This was attributed, in part, to the medium components remaining after the cell wash [26], in agreement with the protein concentrations we measured 5 min after the start of filtration (Figure 5).

**Figure 8:**
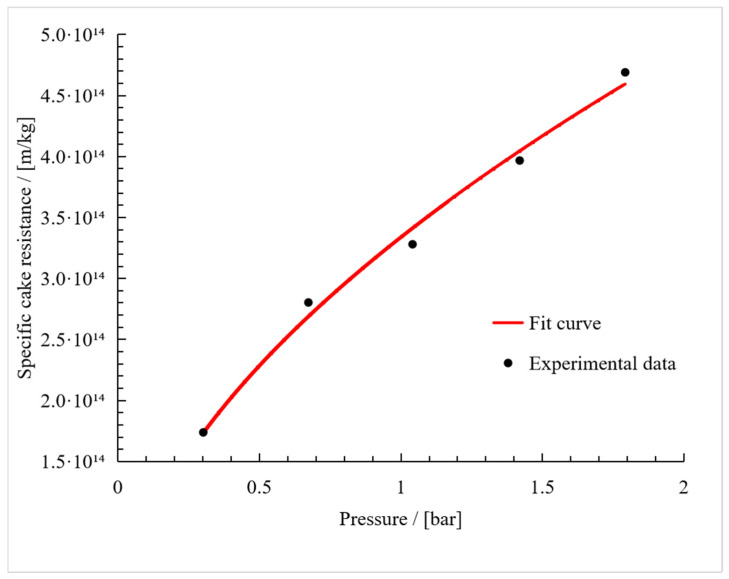
Fit of the specific cake resistance plotted against the pressure over the cake (R^2^ = 0.99).

The first calculation of the compressibility index, based on Equation (8), resulted in a value of 0.56. This was used in Equation (6) to generate the fit curve shown in Figure 8. The compressibility index of the fit curve was 0.55 ± 0.04, and the resistance coefficient was 3.33·10^14^ ± 0.06·10^14^ m^2^·s^2^·kg^−2^. This is comparable to the compressibility indices of 0.5–1.7 reported for *E. coli*, depending on the membrane type and ionic strength [38]. Furthermore, the compressibility indices of rod-shaped *Bacillus circulans* (n = 1.0), *Rhodpseudomonas spheroides* (n = 0.88), and *E. coli* (n = 0.79) were compared with the ellipsoid cells of *Saccharomyces cerevisiae* (n = 0.45) and *Micrococcus glutamicus* (n = 0.31) during dead-end filtration [39]. The compressibility index of *V. natriegens* falls between these two sets of values, perhaps because cross-flow filtration produces a different cake structure based on the oriented stacking of rod-like cells rather than random packing [28]. For n < 1, the flux can be increased by increasing pressure; however, the cake resistance increases faster than increasing TMP when n > 1, leading to a critical flux at a certain TMP. Further increasing TMP does not gain more flux. This is critical to the process design and selection of process parameters.

### 3.3. Application of Hermia’s Laws

**Figure 9:**
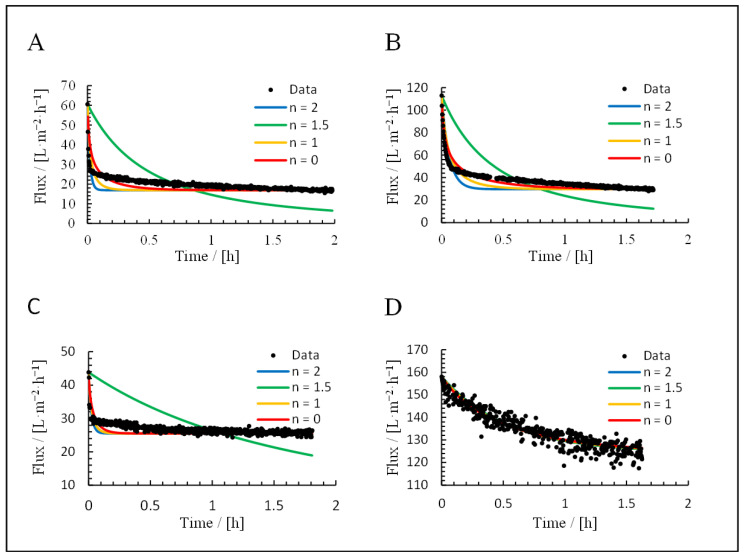
Filtration fit curves using Hermia’s adapted laws: (**A**) culture broth, (**B**) = cells, (**C**) = supernatant, and (**D**) = medium.

**Figure 10:**
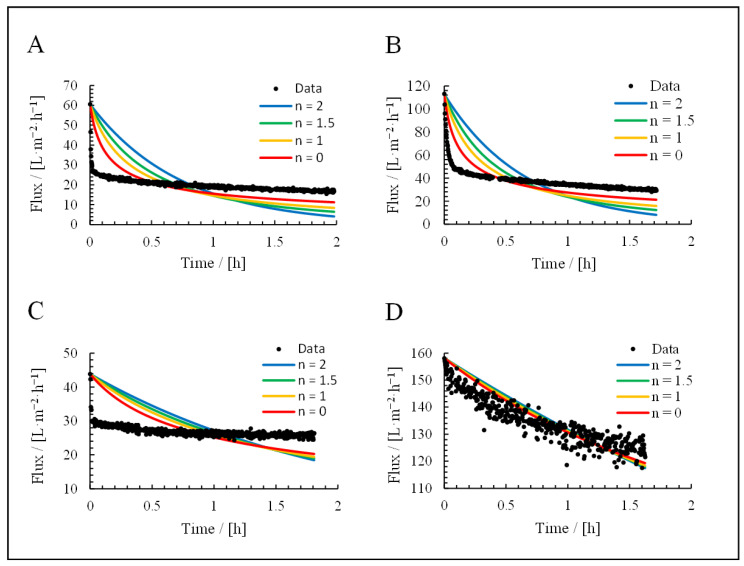
Filtration fit curves using Hermia’s non-adapted laws: (**A**) culture broth, (**B**) = cells, (**C**) = supernatant, and (**D**) = medium.

The permeate curves of each filtration were fitted using Hermia’s laws for cross-flow filtration (Figure 9) and dead-end filtration (Figure 10). The resulting R^2^ values are listed in Table 3 and Table 4. The feeds were the culture broth, cell-free supernatant, cells in NaCl, and cultivation medium.

For the culture broth, the calculated R^2^ values were contradictory. For the fitted formulas, the best model was the standard blocking model (R^2^ = 0.74), but this was not supported by the appearance of the fit curve. Furthermore, only 14% of the resistance was reversible, which would rule out complete blocking and standard blocking as dominant fouling mechanisms according to Figure 1. The application of Hermia’s non-adapted laws improved the fit for the cake filtration model (R^2^ = 0.88). This is the highest value we observed and, therefore, represents the best-fitting model.

For the cell suspension, the best fit was the cake filtration model based on the adapted Hermia’s laws (R^2^ = 0.92), although the non-adapted laws also yielded a high value (R^2^ = 0.88). This was supported by the appearance of the fit curves. The irreversible portion of fouling (29%) was also consistent with these conclusions.

For the supernatant, the calculated R^2^ values were again contradictory. The reversible fouling share (89%) excluded standard and complete blocking as dominant fouling mechanisms. Nevertheless, standard blocking resulted in the best fit for the adapted Hermia’s laws (R^2^ = 0.58), but this was not consistent with the visual appearance of the fit curve. The equation with the best fit using the non-adapted laws was cake filtration (R^2^ = 0.71).

For the medium, the best fit was cake filtration based on the adapted Hermia’s laws (R^2^ = 0.88), although the non-adapted laws delivered similar results. This was supported by the visual appearance of the fit curve and the reversible fouling share of 92%. Based on a visual comparison, the adapted formulas, therefore, provide a more suitable fit in all applications.

## 4. Conclusions

The results of the resistance in series model were conclusive. The individual resistances of the culture components were comparable to those of the culture broth. The instability of the cells in NaCl is an important finding that must be taken into account for future applications, and it should lead to process adaptations (especially shorter filtration times). The method developed to determine the compressibility index during cross-flow filtration is simple and will allow the compressibility index to be determined for other organisms with little effort. We found that the filtration time and feed composition were important parameters that must be investigated to ensure reliable results. The data will be useful to predict the filtration behavior of *V. natriegens*. However, factors such as a change in overflow velocity could limit the accuracy of such predictions and should be investigated in more detail. The application of Hermia’s laws revealed that there is no dominant fouling mechanism during the filtration of the culture broth. This was anticipated because the culture broth is a complex mixture of cells, proteins, and salts. Even the separation of the culture broth into different components did not lead to clear results other than in the case of the cell suspension, where cake filtration was the dominant mechanism. Our data also show that the models have limited applicability when using biological feed streams. Factors such as cell lysis, metabolism, and associated temporal changes in feed composition require more complex models. Nevertheless, we have provided a starting point for the analysis of fouling during the cross-flow filtration of *V. natriegens* cultures, expanding our knowledge of the specific challenges and potential solutions.

## Figures and Tables

**Table 1:** Hermia’s laws for dead-end filtration.

Mechanism	*n*	Equation	
Complete blocking	2	$J=J_{0}\cdot exp(-k\cdot J_{0}\cdot t)$	(12)
Standard blocking	1.5	$J=\frac{J_{0}}{{\left(1+J_{0}\cdot k\cdot t\right)}^{2}}$	(13)
Intermediate blocking	1	$J=\frac{J_{0}}{1+J_{0}\cdot k\cdot t}$	(14)
Cake deposition	0	$J=\frac{J_{0}}{{\left(1+J_{0}\cdot k\cdot t\right)}^{0.5}}$	(15)

**Table 2:** Hermia’s laws for cross-flow filtration.

Mechanism	*n*	Equation	
Complete blocking	2	$J=J_{ss}+(J_{0}-J_{ss})\cdot exp(-k\cdot t)$	(17)
Standard blocking	1.5	$\frac{1}{J^{0.5}}=\frac{1}{J_{0}^{0.5}}+k\cdot t$	(18)
Intermediate blocking	1	$k\cdot t=\frac{1}{J_{ss}}\cdot \ln\left(\frac{J}{J_{0}}\cdot \frac{J_{0}-J_{ss}}{J\cdot J_{ss}}\right)$	(19)
Cake deposition	0	$k\cdot t=\frac{1}{J_{ss}^{2}}\left(\ln\left(\frac{J_{0}}{J}\cdot \frac{J_{0}-J_{ss}}{J-J_{ss}}\right)\right)-J_{ss}\cdot \left(\frac{1}{J}-\frac{1}{J_{0}}\right)$	(20)

**Table 3:** R^2^ values representing fits of Hermia’s laws adapted for cross-flow filtration (the highest values, implicating the best of the fits, are underlined).

Fit Model	R^2^ of the Fit			
	Culture Broth	Cells	Supernatant	Medium
Complete blocking	0.53	0.81	0.50	0.88
Standard blocking	0.74	0.74	0.58	0.88
Intermediate blocking	0.61	0.88	0.53	0.88
Cake filtration	0.72	0.92	0.51	0.88

**Table 4:** R^2^ values representing fits of Hermia’s laws for dead-end filtration (the highest values, implicating the best of the fits, are underlined).

Fit Model	R^2^ of the Fit			
	Culture Broth	Cells	Supernatant	Medium
Complete blocking	0.71	0.69	0.56	0.86
Standard blocking	0.74	0.74	0.58	0.87
Intermediate blocking	0.31	0.14	0.30	0.87
Cake filtration	0.88	0.88	0.71	0.88

## Data Availability

The original contributions presented in this study are included in this article. Further inquiries can be directed to the corresponding author.
